# Antagonistic Effect of a Cytoplasmic Domain on the Basal Activity of Polymodal Potassium Channels

**DOI:** 10.3389/fnmol.2018.00301

**Published:** 2018-09-04

**Authors:** Ismail Ben Soussia, Frank S. Choveau, Sandy Blin, Eun-Jin Kim, Sylvain Feliciangeli, Franck C. Chatelain, Dawon Kang, Delphine Bichet, Florian Lesage

**Affiliations:** ^1^Université Côte d’Azur, INSERM, Centre National de la Recherche Scientifique, Institut de Pharmacologie Moléculaire et Cellulaire, Labex ICST, Valbonne, France; ^2^Department of Physiology, College of Medicine, Institute of Health Sciences, Gyeongsang National University, Jinju, South Korea

**Keywords:** potassium channel, resting membrane potential, excitability, PIP2 – phosphatidylinositol-4, 5-bisphosphate, structure function analysis

## Abstract

TREK/TRAAK channels are polymodal K^+^ channels that convert very diverse stimuli, including bioactive lipids, mechanical stretch and temperature, into electrical signals. The nature of the structural changes that regulate their activity remains an open question. Here, we show that a cytoplasmic domain (the proximal C-ter domain, pCt) exerts antagonistic effects in TREK1 and TRAAK. In basal conditions, pCt favors activity in TREK1 whereas it impairs TRAAK activity. Using the conformation-dependent binding of fluoxetine, we show that TREK1 and TRAAK conformations at rest are different, and under the influence of pCt. Finally, we show that depleting PIP_2_ in live cells has a more pronounced inhibitory effect on TREK1 than on TRAAK. This differential regulation of TREK1 and TRAAK is related to a previously unrecognized PIP_2_-binding site (R329, R330, and R331) present within TREK1 pCt, but not in TRAAK pCt. Collectively, these new data point out pCt as a major regulatory domain of these channels and suggest that the binding of PIP_2_ to the pCt of TREK1 results in the stabilization of the conductive conformation in basal conditions.

## Introduction

TWIK-related K^+^ channels (TREK1, TREK2, and TRAAK) form a subclass of two-pore-domain K^+^ channels (K_2P_), which generate inhibitory leak currents. They are polymodal channels that respond to different signals including membrane stretch, temperature and bioactive lipids. TREK and TRAAK channels are widely expressed in the central and peripheral nervous systems where they play critical roles in pain perception and neuroprotection as well as in anesthesia (Heurteaux et al., 2004; Noel et al., 2011). These channels are homo or heterodimers of subunits with four membrane-spanning domains (M1 to M4), two pore-forming domains (P1 and P2) and a long cytoplasmic C-ter (Enyedi and Czirjak, 2010; Noel et al., 2011; Feliciangeli et al., 2015). Unlike TRAAK, TREK1 and TREK2 have protein partners regulating their channel activity [AKAP150 (Sandoz et al., 2006, 2008), PLD2 (Comoglio et al., 2014)] or their cellular trafficking [MAP2 and POPEYE (Schindler et al., 2016)]. Another major difference is their activity level in basal conditions. In the same expression conditions, TREK1 and TREK2 yield larger currents than TRAAK (Levitz et al., 2016), although these three channels have a similar unitary conductance in physiological conditions (Fink et al., 1998; Patel et al., 1998; Simkin et al., 2008). Two different cytoplasmic regions are known to control current amplitude. The first one is the N-ter that modulates unitary conductance and open probability (Simkin et al., 2008; Thomas et al., 2008). The second is the C-ter that is required for mechano, lipid-, and thermosensitivity (Maingret et al., 2000a; Chemin et al., 2005, 2007; Bagriantsev et al., 2012) as well as for phosphorylation following activation of G protein-coupled receptors by neurotransmitters (Patel et al., 1998). TREK1 mutants truncated in their C-ter are resistant to chemical activation by arachidonic acid (AA) and anesthetics (Patel et al., 1998; Maingret et al., 2000b). Similarly, deletion of the C-ter of TREK2, or substitution with that of TASK3, another K_2P_ channel, abolished the free fatty acid- and pressure sensitivity of the channel (Kim et al., 2001b). However, the replacement of the C-ter of TRAAK with that of TASK1 and TASK3 did not modify the sensitivity of the mutated channel to pressure and AA (Kim et al., 2001a), suggesting that this domain does not play the same role in TREK and TRAAK channels. The recent crystallographic 3-D structures of TREK1 (Lolicato et al., 2014, 2017), TREK2 (Dong et al., 2015) and TRAAK (Brohawn et al., 2012) suggest different conformational states with no firm consensus about the structure of the active open state. The structure of TREK2 bound to its inhibitor fluoxetine suggests that conformational changes modulate channel activity (Dong et al., 2015). TREK2 activation by AA and mechanical stretch would involve conversion between these states through movement of the pore-lining helices. A so-called “down" state would be poorly conductive whereas an “up" state would be more conductive. Fluoxetine would access its binding site only when the channel is in the “down" state.

Here, we looked for the structural element of TREK1 and TRAAK that may explain their marked difference in the currents produced in basal conditions. By a combination of deletions and domain swapping, we first showed that the cytoplasmic domain located immediately after the last membrane-spanning segment (the proximal C-ter domain, pCt) is directly responsible for this difference. It acts as an inhibitor in TRAAK and as an activator in TREK1. We next probed the possibility that this domain may influence the conformational state of the channels in basal conditions. So, we used the ability of fluoxetine to bind to the “down” state of TREK2. As previously shown for TREK2, we found that TREK1 is blocked by fluoxetine and is therefore in the “down” conformation in basal conditions, but that activation by AA induced a switch of conformation from the “down” to the “up.” A surprising result is that TRAAK, which is not sensitive to fluoxetine at rest becomes sensitive once activated by AA, demonstrating that TRAAK also contains a binding site for fluoxetine. Furthermore, swapping pCt between TREK1 and TRAAK exchanges this sensitivity, suggesting that pCt influences the conformational state of TREK1 and TRAAK in an opposite manner. Finally, we tested the potential role of phosphatidylinositol-4, 5-bisphosphate (PIP_2_) on channel activity. Previous studies have shown that this lipid, located in the inner leaflet of the plasma membrane, modulates TREK1 and TREK2 channels through an interaction with basic residues within pCt (Chemin et al., 2005; Sandoz et al., 2011; Woo et al., 2016, 2018; Lolicato et al., 2017). We used a voltage-sensitive phosphatase from *Danio rerio* (VSP), which dephosphorylates nearly all the PIP_2_ in the membrane (Okamura et al., 2009), to evaluate its role in the gating of TREK1 and TRAAK. We found that TREK1 and TRAAK have different responses to PIP_2_ depletion, suggesting different affinities. This difference of affinity is due to a second PIP_2_ action site (R329, R330, R331) within TREK1 that is not conserved in TRAAK. Altogether, these results suggest that the difference in channel activity at rest between TREK1 and TRAAK is due to the unique capacity of the pCt to bind PIP_2_ and to promote an active conformational state in TREK1. This site is absent in TRAAK resulting in a poorly conductive state and low channel activity.

## Results

### A Discrete Domain Controls TREK1 and TRAAK Current Amplitudes in Basal Conditions

**FIGURE 1:**
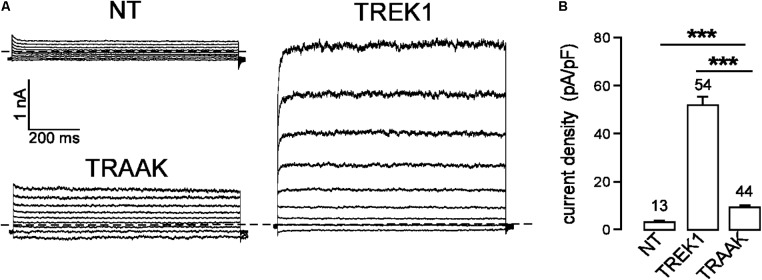
TREK1 and TRAAK currents in HEK293 cells. **(A)** Representative whole-cell currents from non-transfected cells (NT) and cells transfected with TREK1 and TRAAK channels. Voltage steps were applied from –100 to 60 mV in 20 mV increments from a holding potential of –80 mV. The dotted lines indicate zero current. **(B)** Current densities at 0 mV. Data are presented as mean ± SEM. ^∗∗∗^*p* < 0.001, the number of cells is indicated (Student’s *t*-test).

**FIGURE 2:**
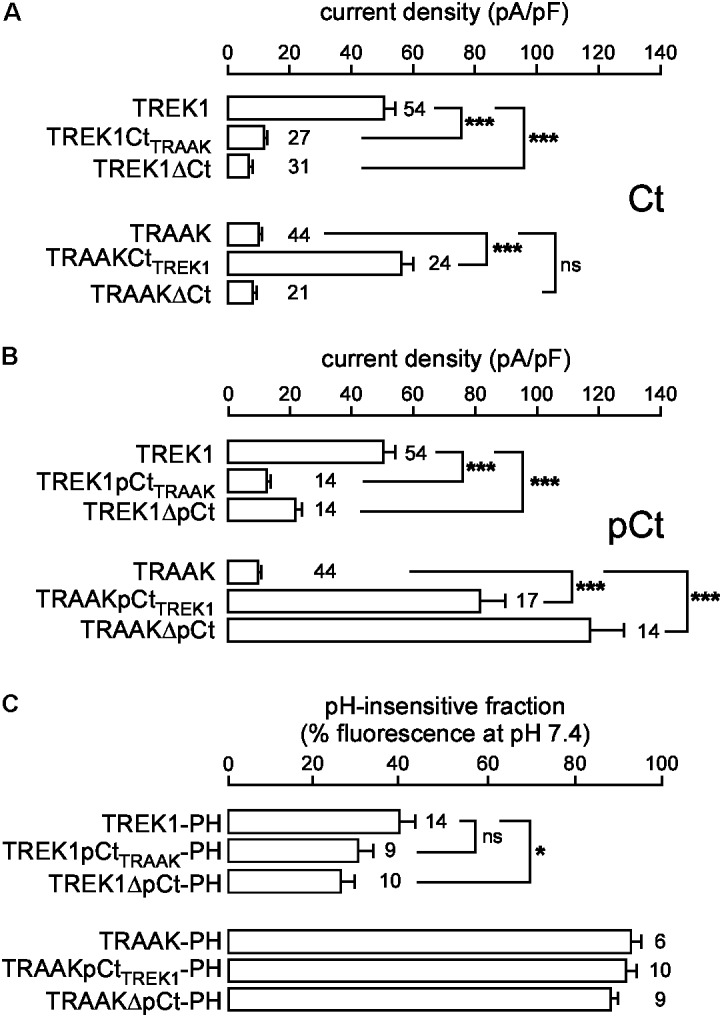
Proximal C-ter domain (pCt) modulates TREK1 and TRAAK current amplitudes but not their cellular distribution. **(A,B)** Current densities at 0 mV of HEK cells expressing wild type or mutated channels. Ct, full cytoplasmic C-ter **(A)**. pCt, proximal cytoplasmic C-ter **(B)**. **(C)** % of the fluorescence sensitive to an extracellular acidification (pH 6) of live HEK cells expressing pHluorin-tagged channels. This % corresponds to the fraction of channels expressed at the plasma membrane. **(A–C)** Data are presented as mean ± SEM. ^∗^*p* < 0.05, ^∗∗∗^*p* < 0.001, and ns, not significant. The number of cells is indicated (one-way Anova with Tukey’s test).

In the same expression conditions, TREK1 produced 6-time more current than TRAAK (51.9 ± 3.6 pA/pF vs. 9.0 ± 0.7 pA/pF at 0 mV, **Figure 1**). To investigate a potential role of the cytoplasmic C-ter (Ct), we first deleted this region in both channels. This deletion strongly decreased TREK1 from 51.9 ± 3.6 pA/pF to 7.2 ± 0.8 pA/pF at 0 mV, but had no significant effect on TRAAK (9.2 ± 0.8 pA/pF for WT vs. 8.4 ± 0.8 pA/pF for the deleted mutant, **Figure 2A**). The replacement of the C-ter of TRAAK by the C-ter of TREK1 induced a 10-fold increase of TRAAK current density whereas the reverse swapping induced a strong decrease of TREK1 current (**Figure 2A**). We next restricted the deletion to the proximal part of the cytoplasmic C-ter (pCt, **Figure 2B**). The deletion of pCt from W295 to A343 in TREK1, and from W256 to P302 in TRAAK promoted a decrease of the current density of TREK1 (from 51.9 ± 3.6 pA/pF to 21.7 ± 2.3 pA/pF) and an increase of that of TRAAK (from 9.2 ± 0.8 pA/pF to 117.2 ± 11.1 pA/pF). The swapping of TREK1 and TRAAK pCts also drastically reduced current density of TREK1pCt_TRAAK_ (from 51.9 ± 3.6 pA/pF for TREK1 to 12.6 ± 1.3 pA/pF for TREK1pCt_TRAAK_). In contrast, the reverse substitution (TRAAKpCt_TREK1_) led to a 10-fold current density increase. Interestingly, TRAAKpCt_TREK1_ had a current density similar to TREK1 (56.0 ± 4.1 pA/pF vs. 51.9 ± 3.6 pA/pF). These results show that pCt is involved in the difference of current amplitudes between TREK1 and TRAAK. TRAAK pCt acts as an inhibitor, whereas TREK1 pCt acts as an activator.

To rule out the possibility that these differences in the current density could be due to changes in the cellular distribution of the channels, we fused pHluorin to their C-ter, and used the pH-sensitivity of pHluorin (PH) to estimate the fraction of protein expressed at the plasma membrane. pHluorin is a modified green fluorescent protein that is fluorescent at pH 7.4 but not at pH 6. An additional membrane-spanning segment was added between the channel and pHluorin, to expose pHluorin to the extracellular medium (**Supplementary Figure S3A**). The quantity of pHluorin-tagged channels present at the plasma membrane of live transfected cells was estimated by shifting the value of the extracellular pH from 7.4 to 6 and by quantifying the percentage of fluorescence sensitive to this acidic shift (**Figure 2C** and **Supplementary Figure S3C**). Fusion of pHluorin did not affect current densities (**Supplementary Figure S2**) or cellular distributions (**Supplementary Figures S3B,C**) of TREK1 and TRAAK. The fluorescence produced by TREK1-PH at the cell perimeter vanished at pH 6, demonstrating that TREK1 is mainly expressed at the plasma membrane. TREK1ΔpCt-PH and TREK1pCt_TRAAK_-PH exhibited the same cell distribution, mainly at the cell surface (**Figure 2C** and **Supplementary Figure S3C**). The fluorescence produced by TRAAK-PH as well as TRAAKΔpCt-PH and TRAAKpCt_TREK1_-PH was almost the same at pH 7.4 and pH 6, (**Figure 2C** and **Supplementary Figure S3C**) suggesting that these proteins are mainly located in neutral intracellular compartments. These results demonstrate that swapping pCt between TREK1 and TRAAK has no major effect on their cellular trafficking. Altogether, these results demonstrate that pCt directly controls TREK1 and TRAAK activities, without affecting their distribution in the cells.

As recently reported, TREK1, TREK2, and TRAAK assemble to form active heterodimers (Blin et al., 2016; Lengyel et al., 2016; Levitz et al., 2016). Each TREK1-TRAAK heterodimer contains two pCt, one from TREK1 and the other one from TRAAK. How will these domains with antagonistic actions regulate channel activity in the heterodimer? To address this question, we expressed different covalent dimers in which subunits were fused in tandem. TRAAK-TREK1 heterodimers produced more current than TRAAK-TRAAK and TREK1-TREK1 homodimers (**Supplementary Figure S1**). At 0 mV, the current densities were 52.2 ± 8.3 pA/pF for TREK1-TREK1, 20.3 ± 2.3 pA/pF for TRAAK-TRAAK and 113.7 ± 15.3 pA/pF for TRAAK-TREK1 (**Supplementary Figures S1A–C,F**). The replacement of the pCt of TREK1 by that of TRAAK resulted in a dramatic reduction of the current density (28.1 ± 4.7 pA/pF for TRAAK-TREK1pCt_TRAAK_, **Supplementary Figures S1D,F**). Interestingly, TRAAK-TREK1pCt_TRAAK_ current density was similar to TRAAK-TRAAK. Conversely, the substitution of the pCt of TRAAK by that of TREK1 strongly increased the current (219.3 ± 36.2 pA/pF for TRAAKpCt_TREK1_-TREK1, **Supplementary Figures S1E,F**). These results demonstrate that the pCt of TREK1 plays a dominant activating role over the inhibiting pCt of TRAAK.

### pCt Modulates TREK1 and TRAAK Single-Channel Properties

**FIGURE 3:**
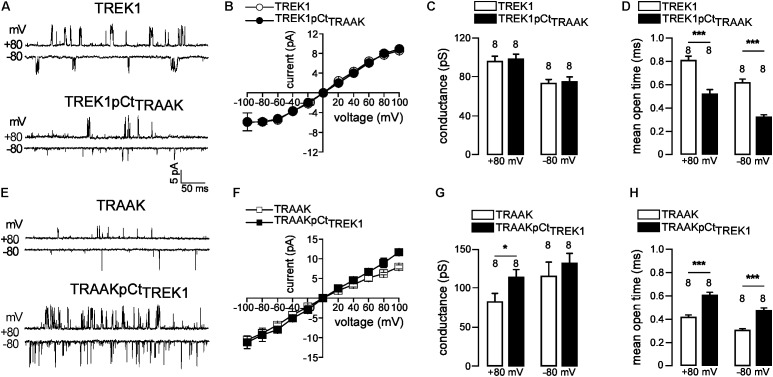
pCt modulates TREK1 and TRAAK single-channel properties. **(A,E)** Single-channel recordings at +80 and –80 mV, from HEK cells expressing wild-type and mutated channels. Pipette and bath solutions contained 150 mM KCl. **(B,F)** Single channel current–voltage relationships obtained from one level opening current in symmetrical 150 mM KCl. **(C,G)** Unitary conductances at +80 and –80 mV. **(D,H)** Mean open time obtained from channel opening recorded at +80 mV and –80 mV. **(C,D,H,G)**, Data are presented as mean ± SEM, the number of patches is indicated, ^∗^*p* < 0.05, ^∗∗∗^*p* < 0.001, (Mann–Whitney test).

If pCt directly affects channel activity then this should be visible on the single channel properties of TREK1 and TRAAK. To address this question, we compared the single-channel properties of WT (TREK1 and TRAAK) and mutant (TREK1pCt_TRAAK_ and TRAAKpCt_TREK1_) channels in the same expression conditions. TREK1 and TRAAK displayed typical single-channel openings at +80 and −80 mV under cell-attached patch mode (**Figure 3A**). TREK1pCt_TRAAK_ displayed shorter open time and lower frequency than TREK1, without changes in amplitude. In contrast, TRAAKpCt_TREK1_ showed longer open time, higher frequency, and bigger amplitude at +80 mV compared to TRAAK (**Figure 3E**). The current-voltage (IV) relationship of TREK1pCt_TRAAK_ was similar to that of TREK1 showing outward rectification (**Figure 3B**), whereas the IV relationship of TRAAKpCt_TREK1_ was slightly different from TRAAK at positive potentials (**Figure 3F**). TRAAK and TRAAKpCt_TREK1_ exhibited a weak inward rectification and a linear IV relationship, respectively. The unitary conductance of TREK1 and TREK1pCt_TRAAK_ were similar: 96.3 ± 5.2 pS and 98.6 ± 4.8 pS at +80 mV, and 73.8 ± 4.0 pS and 75.1 ± 4.8 pS at −80 mV, respectively (**Figure 3C**). In contrast, the unitary conductance of TRAAKpCt_TREK1_ was significantly higher than that of TRAAK at +80 mV (114.0 ± 9.2 pS vs. 82.9 ± 15.2 pS), but not at −80 mV (132.9 ± 12.6 pS vs. 116.0 ± 17.4 pS, **Figure 3G**). Finally, the mean open time at +80 mV of TREK1pCt_TRAAK_ was significantly decreased compared to TREK1 (0.52 ± 0.04 ms vs. 0.80 ± 0.03 ms) whereas that of TRAAKpCt_TREK1_ was increased compared to TRAAK (0.60 ± 0.02 ms vs. 0.41 ± 0.02 ms, **Figures 3D,H**). Similar results were obtained at −80 mV. These results demonstrate that pCt modulates the gating of TREK1 and TRAAK, promoting more activity in TREK1 than in TRAAK.

### pCt Is Physically Coupled to the Channel Gate

**FIGURE 4:**
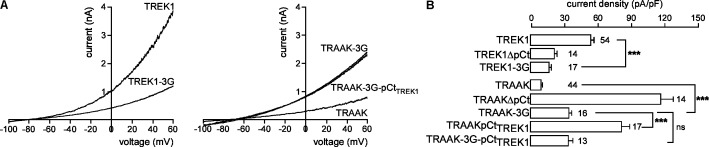
Effect of decoupling pCt and M4 on TREK1 and TRAAK. **(A)** Representative whole-cell recordings of HEK cells expressing wild-type or mutated channels. Voltage ramps were applied from –100 to 60 mV from a holding potential of –80 mV. **(B)** Current densities at 0 mV. Data are presented as mean ± SEM. ^∗∗∗^*p* < 0.001, and ns, not significant. The number of cells is indicated (one-way Anova with Tukey’s test).

Previous studies have shown that the membrane-spanning helix M4 and the cytosolic C-ter are functionally coupled in K_2P_ channels (Miller and Long, 2012; Dong et al., 2015). An allosteric coupling between pCt and the selectivity filter (SF) has been described in TREK2. A movement of M4 facilitates this coupling (Zhuo et al., 2016). To investigate the possibility that coupling is necessary for pCt action in TREK1 and TRAAK, we modified the M4/pCt junction by replacing residues 292 to 294 by glycines in TREK1 to produce TREK1-3G, and residues 253 to 255 in TRAAK to produce TRAAK-3G. This repeat of glycine residues introduces structural flexibility and is expected to prevent transmission of conformational changes between pCt, and the SF/M4 module. TREK1-3G current density is much smaller than that of TREK1 (17.2 ± 1.7 pA/pF vs. 51.9 ± 3.6 pA/pF, **Figures 4A,B**), whereas that of TRAAK-3G is much higher than that of TRAAK (34.8 ± 3.6 pA/pF vs. 9.2 ± 0.8 pA/pF, **Figure 4B**). Furthermore, the 3G mutation abolished the activating effect of TREK1 pCt on TRAAK as TRAAK-3GpCt_TREK1_ current density is similar to that of TRAAK-3G (32.5 ± 6.4 pA/pF vs. 34.8 ± 3.6 pA/pF, **Figure 4B**). Collectively, these data demonstrate that pCt and SF/M4 are mechanically coupled in TREK1 and TRAAK. This coupling is necessary for the activating property of TREK1 pCt and the inhibiting effect of pCt TRAAK.

### pCt Controls Channel Conformation

**FIGURE 5:**
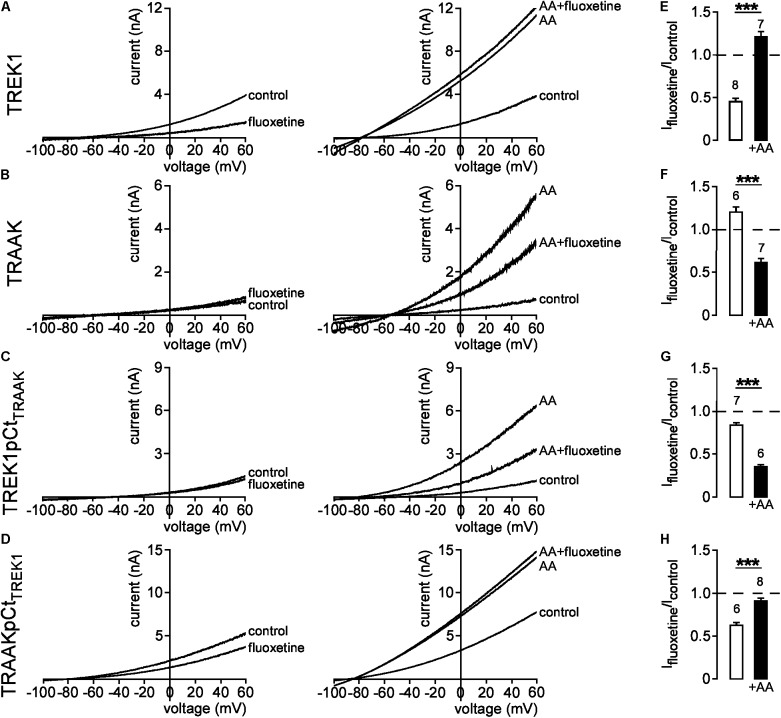
State-dependent inhibitory effect of fluoxetine. **(A–D)** Representative patch-clamp recordings from HEK cells expressing wild-type or mutated channels in the presence or absence of 10 μM fluoxetine on basal currents (left panel) and on currents stimulated by 10 μM AA (right panel). (**E–H)** Fraction of fluoxetine-sensitive current in the absence or presence of 10 μM AA. Data are presented as mean ± SEM. ^∗∗∗^*p* < 0.001, the number of cells is indicated (Student’s *t*-test).

We next evaluated the effect of pCt on the conformation of TREK1 and TRAAK. Previous studies have shown that fluoxetine inhibits TREK1 and TREK2, but not TRAAK (Kennard et al., 2005; Heurteaux et al., 2006; Thummler et al., 2007). This inhibition depends on the conformation state of the channel (Dong et al., 2015). Fluoxetine binds to TREK2 only when the channel is in the so-called “down” conformation. The conversion to an “up” conformation by application of AA stimulates the channel that becomes insensitive to fluoxetine. Consistent with these previous studies, fluoxetine inhibited TREK1 in basal conditions (I_fluoxetine_/I_control_ = 0.45 ± 0.04, **Figure 5A**, left panel and **Figure 5E**), but not TREK1 stimulated by AA (I_fluoxetine_/I_control_ = 1.21 ± 0.06, **Figure 5A**, right panel and **Figure 5E**). This behavior is similar to the behavior of TREK2 suggesting that TREK1 is in a fluoxetine-sensitive “down” conformation at rest and in a fluoxetine-resistant “up” conformation when stimulated. As previously described, TRAAK was resistant to fluoxetine at rest (I_fluoxetine_/I_control_ = 1.20 ± 0.07, **Figure 5B**, left panel and **Figure 5F**). However, here we show that TRAAK was inhibited by fluoxetine after stimulation by AA (I_fluoxetine_/I_control_ = 0.62 ± 0.04, **Figure 5B** right panel and **Figure 5F**). This unexpected result demonstrates that as TREK1 and TREK2, TRAAK contains a binding site for fluoxetine but that this site is accessible only when the channel is stimulated. These results suggest that if TREK1 and TRAAK share the same overall conformations, TRAAK is in the “up” state in basal conditions and in the “down” conformation when activated.

Kennard et al. (2005) have shown that residue E306 within pCt may be involved in the inhibition of TREK1 by fluoxetine. Substitution of E306 by an alanine reduces fluoxetine inhibition. This inhibition has been suggested to impair the interaction of the cytoplasmic C-ter with the plasma membrane, hence promoting channel activation (Chemin et al., 2005; Sandoz et al., 2011). Therefore, we tested the hypothesis that pCt may influence TREK1 sensitivity to fluoxetine. We found that TREK1pCt_TRAAK_ behaved like TRAAK. In basal conditions, fluoxetine had only a minor effect on the mutant channel (I_fluoxetine_/I_control_ = 0.83 ± 0.04, **Figure 5C**, left panel and **Figure 5G**). However, AA-induced TREK1pCt_TRAAK_ activation made the channel sensitive to fluoxetine (I_fluoxetine_/I_control_ = 0.35 ± 0.03) (**Figure 5C**, right panel and **Figure 5G**). Unlike TREK1pCt_TRAAK_, TRAAKpCt_TREK1_ was sensitive to fluoxetine in its resting state (I_fluoxetine_/I_control_ = 0.63 ± 0.03) (**Figure 5D**, left panel and **Figure 5H**). Fluoxetine had no effect on AA-activated TRAAKpCt_TREK1_ (I_fluoxetine_/I_control_ = 0.92 ± 0.03) (**Figure 5D**, right panel and **Figure 5H**). These results show that pCt controls the sensitivity to fluoxetine in TREK1 and TRAAK. Taken together, these data show that pCt directly modulates the conformational state of these channels, affecting their activity levels and their sensitivity to fluoxetine.

### pCt Function of TREK1 Is Related to Its Affinity for PIP_2_

**FIGURE 6:**
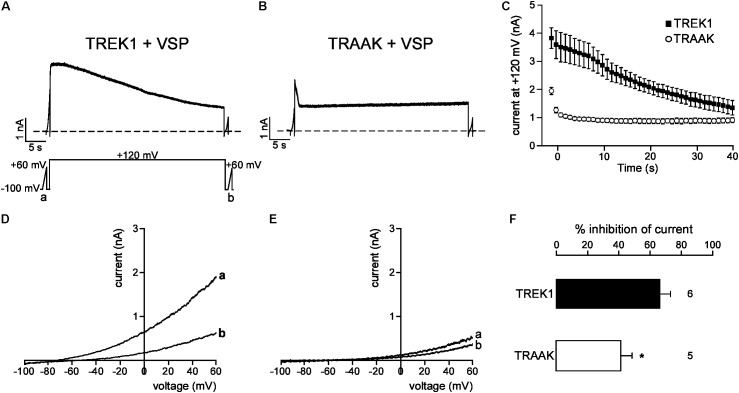
PIP_2_ depletion inhibits TREK1 and TRAAK currents. **(A,B)** Representative perforated patch-clamp recordings from HEK cells co-expressing VSP and TREK1 **(A)** or TRAAK **(B)**. **(C)** Average currents measured at +120 mV during VSP-induced PIP_2_ depletion. **(D,E)**, TREK1 **(D)** and TRAAK **(E)** currents before (a) and after (b) the +120 mV/40 s depolarizing pulse. **(F)** Current inhibition (%) of TREK1 and TRAAK at 0 mV. Data are presented as mean ± SEM. ^∗^*p* < 0.05, the number of cells is indicated (Student’s *t*-test).

**FIGURE 7:**
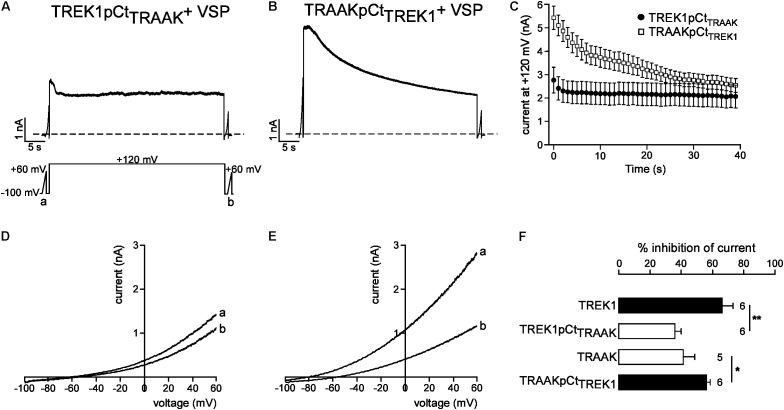
Role of pCt in TREK1 and TRAAK differential sensitivities to PIP_2_. **(A,B)** Representative perforated patch-clamp recordings from HEK cells co-expressing VSP and TREK1pCt_TRAAK_ **(A)** and TRAAK pCt_TREK1_ **(B)**. **(C)** Average currents measured at +120 mV during VSP-induced PIP_2_ depletion. **(D,E)** TREK1pCt_TRAAK_ **(D)** and TRAAKpCt_TREK1_ **(E)** currents before (a) and after (b) the +120 mV/40 s depolarizing pulse. **(F)** Current inhibition (%) of wild-type and mutated channels at 0 mV. Data are presented as mean ± SEM. ^∗^*p* < 0.05, ^∗∗^*p* < 0.01, the number of cells is indicated (Student’s *t*-test).

We next studied the molecular mechanism by which TREK1 pCt and TRAAK pCt could exert opposite actions. The cytoplasmic region adjacent to M4 has previously been shown to interact with PIP_2_ in TREK1 (Chemin et al., 2005; Sandoz et al., 2011; Lolicato et al., 2017). Acidification acting on E306 or phosphorylation of adjacent serine residues affect the electrical charge of this region and its interaction with the plasma membrane, leading to a modification of channel conformation and activity. The current hypothesis is that PIP_2_ is also involved in this interaction. To test the role of PIP_2_ in the action of TREK1 and TRAAK pCt, we measured current inhibition and time course of current decay after PIP_2_ depletion. To do so, we used a voltage-sensitive phosphatase from *Danio rerio* (VSP) (Okamura et al., 2009). Upon strong membrane depolarization, this enzyme is activated and dephosphorylates PIP_2_ to generate PIP. Voltage ramps from −100 mV to +60 mV were applied to record baseline channel current (ramp a), then a large depolarizing pulse (+120 mV for 40 s) was applied to activate VSP-induced PIP_2_ depletion, followed by a second ramp protocol (ramp b) to measure the effect of this depletion on the current (**Figure 6A**). In control cells expressing no VSP, TREK1, and TRAAK currents were not affected by the large depolarizing pulse (**Supplementary Figure S4**). In contrast, in cells expressing VSP, TRAAK and TREK1 currents in ramp b were smaller than those in ramp a (**Figures 6D,E**), suggesting that both channels are sensitive to PIP_2_. However, this sensitivity is different. TREK1 current is more reduced than TRAAK current (66.0 ± 7.1% vs. 40.1 ± 7.4% at 0 mV, **Figure 6F**) and its inhibition at +120 mV is much slower (∼40 s, **Figures 6A,C**) than that of TRAAK current (∼5 s, **Figures 6B,C**). These data suggest that both channels are regulated by PIP_2_ but that the affinity of TRAAK for PIP_2_ is low. We then tested the effect of VSP activation on TREK1pCt_TRAAK_ and TRAAKpCt_TREK1_. Replacement of the pCt of TREK1 with that of TRAAK decreased TREK1 sensitivity to PIP_2_ whereas the reverse substitution increased that of TRAAK (**Figure 7**). TREK1pCt_TRAAK_ current is quickly reduced at +120 mV (**Figures 7A,C**) and weakly inhibited compared to TREK1 (36.4 ± 3.7, **Figure 7F**). In contrast, TRAAKpCt_TREK1_ current is slowly reduced at +120 mV (**Figures 7B,C**) and strongly inhibited like TREK1 (56.4 ± 2.3%, **Figure 7F**). These results show that the influence of the pCt in TREK1 and TRAAK is directly related to its affinity for PIP_2_.

**FIGURE 8:**
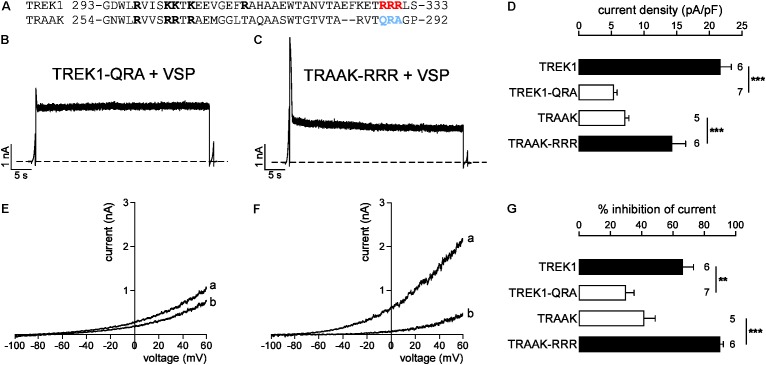
Sensitivity to PIP_2_ is modulated by a cluster of basic residues in pCt. **(A)** Sequence alignment of TREK1 and TRAAK pCt. In red and blue the residues that are swapped in TREK1-QRA and TRAAK-RRR. **(B,C)** Representative perforated patch-clamp recordings from HEK cells co-expressing VSP and TREK1-QRA **(B)** and TRAAK-RRR **(C)**. Same protocol as in **Figures 6A**, **7A**. **(D)** Current density (%) at 0 mV. **(E,F)** TREK1-QRA **(E)** and TRAAK-RRR **(F)** currents before (a) and after (b) the +120 mV/40 s depolarizing pulse. **(G)** Current inhibition (%) at 0 mV. **(D,G)** Data are presented as mean ± SEM. ^∗∗^*p* < 0.01, ^∗∗∗^*p* < 0.001, the number of cells is indicated (Student’s *t*-test).

A previous study has identified a cluster of basic residues (R297, K301, K302, K304, and R311 in bold, **Figure 8A**) in TREK1 as critical for PIP_2_-channel interaction (Chemin et al., 2005). Four of these five positively charged residues (R258, R262, R263, and R265) are conserved in TRAAK suggesting that this basic cluster cannot explain the difference in affinity between TREK1 and TRAAK. A sequence alignment shows that TREK1 pCt contains another cluster of basic residues (R329, R330, R331; in red and in bold) that are not conserved in TRAAK pCt (Q288, R289, and A290; in blue and in bold, **Figure 8A**). To test their implication, we swapped these residues between TREK1 and TRAAK. We found that TREK1-QRA channels behave like TRAAK, with a fast current decay (**Figure 8B**) and a weak inhibition of current following VSP activation (30.2 ± 5.2%, **Figures 8E,G**). Moreover, the QRA mutation significantly decreased TREK1 current density, lowering it down to the same level than that of TRAAK (**Figure 8D**). Conversely, the RRR mutation in TRAAK significantly increased its current density (**Figure 8D**) and its sensitivity to PIP_2_ depletion like in TREK1 (from 40.1 ± 7.4% of inhibition to 89.7 ± 2.2%, **Figures 8F,G**). Together, these results show that a cluster of basic residues (R329, R330, R331) in TREK1 pCt contributes to the binding of PIP_2_. Disruption of this interaction converts high activity TREK1 into a low activity channel as observed for TRAAK. The fast current decay of TRAAK-RRR similar to TRAAK upon VSP activation (**Figure 8C**) shows that other additional residues are most probably involved in the regulation of TRAAK by PIP_2._

Finally, we tested the sensitivity of TRAAK-RRR and TREK1-QRA to fluoxetine. As expected, TREK1-QRA behaves like TRAAK. In basal conditions, fluoxetine had only a minor effect on TREK1-QRA (I_fluoxetine_/I_control_ = 0.81 ± 0.02, **Supplementary Figures S5A,C**). And, like TREK1, TRAAK-RRR was sensitive to fluoxetine in its resting state (I_fluoxetine_/I_control_ = 0.60 ± 0.03, **Supplementary Figures S5D–F**). These results show that QRA residues in TRAAK and the RRR residues in TREK1 play a role in channel sensitivity to fluoxetine, suggesting an effect on the channel conformation.

## Discussion

TREK1, TREK2, and TRAAK ability to convert multiple mechanical and chemical stimuli into finely tuned electrical responses is unique among the K^+^ channels. Which structural changes are induced during this process remains an open and fascinating question. Recent crystal structures of TREK and TRAAK channels provided new insights on the open and closed conformations of these channels (Brohawn et al., 2012, 2014; Miller and Long, 2012; Lolicato et al., 2014; Dong et al., 2015). These channels have been suggested to exist in two main conformations, showing differences in the vertical arrangement of the pore-lining helices: the “up” and “down” conformations. Original structural studies were in apparent disagreement, TRAAK being reported to be open in the “up” state in one study (Brohawn et al., 2014) and in the “down” state in the other (Lolicato et al., 2014). TREK2 closed state was then suggested to correspond to the down state (Dong et al., 2015). More recent studies suggest that for TREK1 and TREK2 both “up” and “down” conformations are conductive (McClenaghan et al., 2016; Lolicato et al., 2017). These structures could not provide any information on the proximal C-ter (pCt) since the crystallized channels were truncated and devoid of their C-ter. Previous studies have shown that this pCt is required for the integration of many stimuli. This domain is the site of action for internal acidification and phosphorylation (Patel et al., 1998; Murbartián et al., 2005), and is indirectly necessary for the action of pressure and temperature (Bagriantsev et al., 2012; Maingret et al., 2000a). Here, we show that pCt directly controls TREK1 and TRAAK current amplitudes even in the absence of stimuli. Surprisingly, pCt has opposite effects in TREK1 and TRAAK: it acts as an inhibitor in TRAAK and as an activator in TREK1. Deletion of this region dramatically decreased TREK1 current amplitude, but increased by nearly 13-fold that of TRAAK. The same effects were observed when the two pCt were swapped. However, this is not due to a change in channel trafficking to the plasma membrane but directly related to modifications of channel properties, mainly mean open time and open probability. Altogether, these new data explain why TRAAK is less active than TREK1 in basal conditions.

Previous studies have suggested that pCt is coupled to the SF in K_2P_ channels through the M4 domain (Miller and Long, 2012; Dong et al., 2015). Mutations at the M4/pCt junction, which decouples the C-ter from the SF, abolishes both C-ter-dependent modulations by membrane potential and temperature (Bagriantsev et al., 2012). Here, we show that the substitution of three residues at the M4/pCt junction of TREK1 and TRAAK with glycines, whose flexibility decouples pCt from SF, rendered the resulting mutant channels much less sensitive to the modulatory effect of pCt. This 3G mutation induced a 3-fold decrease of TREK1 current amplitude and a 4-fold increase of that of TRAAK. Furthermore, the activating effect of TREK1 pCt was abolished when it was expressed in TRAAK-3G mutant. These results demonstrate that pCt/SF coupling controls TREK and TRAAK activity. This also shows that in these channels, the mechanical coupling between pCt and SF is also important in basal conditions. To understand how pCt affects channel conformation and activity, we took advantage of the state-depending binding of fluoxetine. Fluoxetine binds to TREK2 only in the “down” conformation (Dong et al., 2015). Once activated by AA, TREK2 shifts to the “up” conformation and becomes resistant to fluoxetine. Like TREK2, TREK1 is sensitive to fluoxetine in basal conditions and thus has to be in the “down” conformation. Here, we show that if TRAAK is indeed insensitive to fluoxetine as previously described (Thummler et al., 2007), it becomes sensitive to this blocker once stimulated by AA. These opposite behaviors of TRAAK and TREK1 are due to their pCt, since swapping this domain between TREK1 and TRAAK promotes an exchange of their properties regarding fluoxetine sensitivity in the stimulated state. These results suggest that TREK1 would be in a “down” and fluoxetine-sensitive state in basal conditions whereas TRAAK would be in a similar state when stimulated by AA. Because of the inherent limitations of the mutagenesis approach – and even if our interpretations are guided by reference to actual structures-, we cannot firmly conclude in the absence on new 3D-structures. These new data about pCt also show how important it is to get 3D-structures from channels retaining this pCt region to better investigate their functional conformations.

A mechanical coupling between the SF/M4 module and pCt implies that these domains exert physical forces on each other. Replacing TRAAK pCt by TREK1 pCt induces a strong current increase in TRAAK, due to a conformational change of the SF/M4 module as supported by the accessibility of fluoxetine to its binding site. This implies that TREK1 pCt pushes or pulls on the SF/M4 module. The transmission of a force requires the anchoring of pCt to a cell element. Several lines of evidence support the idea that pCt interacts with the plasma membrane. Internal acidification, renders TREK1 pCt less negatively charged by acting on a glutamate residue (E306). This could increase the interaction of pCt with the negatively charged phospholipids of the inner leaflet of the plasma membrane, stimulating channel activity (Honoré et al., 2002; Chemin et al., 2005). Conversely, phosphorylation of serine residues (S333) introduces negative charges, decreasing interaction of pCt with the membrane and channel activity. Data suggest that PIP_2_ could be the negatively charged phospholipid of the plasma membrane involved in its binding to pCt. Exogenous application of PIP_2_ activates TRAAK (Lopes et al., 2005), inhibits TREK2 currents (Zheng et al., 2009; Woo et al., 2016) and has a dual effect on TREK1 (Chemin et al., 2007). However, a prolonged depletion of PIP_2_ induced by a voltage-sensitive phosphatase leads to a decrease of TREK2 currents (Woo et al., 2016). Several positively charged residues (R297, K301, K302, K304, and R311) in the proximal C-ter of TREK1 seems crucial for PIP_2_ interactions (Chemin et al., 2005), a view also supported by the recent crystal structure of this channel (Lolicato et al., 2017). According to the 3D-structures of TREK1 and TRAAK, the positively charged residues can move toward the plasma membrane to promote the conductive conformation (Brohawn et al., 2014; Lolicato et al., 2014; Dong et al., 2015). Consistent with this, a soluble fluorescent binding assay suggested that PIP_2_ might displace basic residues of the pCt at the most 8Å away from the plasma membrane (Cabanos et al., 2017). Here, we show that pCt affects channel conformation by interacting with PIP_2_ in live cells. We used a voltage-sensitive phosphatase (VSP), which dephosphorylates PIP_2_ into PIP. As previously described (Woo et al., 2016), activation of this phosphatase induced first a weak increase of TREK1 current, then a sustained decrease, confirming the dual effect of PIP_2_ on TREK1. Unlike TREK1, TRAAK is quickly inhibited by the depletion of PIP_2_, suggesting that TREK1, and TRAAK have different affinities for PIP_2_. Moreover, the effects of VSP activation on the current amplitude and the current decay suggest that TREK1pCt_TRAAK_ has the same sensitivity for PIP_2_ as TRAAK, whereas TRAAKpCt_TREK1_ has a PIP_2_ affinity similar to that of TREK1. Based on sequence alignment, we identified three positively charged residues (R329, R330, R331) in the pCt of TREK1 that are not all conserved in TRAAK. At the equivalent position in TRAAK, the residues are Q288R289A230, a motif containing only one charge. By swapping these three residues between TREK1 and TRAAK, we found that the QRA mutation strongly decreased TREK1 current amplitude and rendered this channel less sensitive to the depletion of PIP_2_. In contrast, the RRR mutation significantly increased TRAAK current amplitude as well as its sensitivity to PIP_2_. The conclusion is that these basic residues (R329, R330, and R331) contribute to the binding of PIP_2_ binding in TREK1. Its absence in TRAAK may partially explain the PIP_2_-sensitivity and the low current density at rest. Given that these three arginines are conserved in TREK2, we expect them to play a role in the regulation of TREK2 by PIP_2_. However, pCt may not be the sole PIP_2_-binding site. Indeed, PIP_2_ may engage two other sites at inter-subunit junctions: one at the M1-M2-M4 and another at the M2-P2 (Lolicato et al., 2017). Interestingly, such inter-subunit interactions have also been described in the crystal structures of transmembrane domain channels, Kir2.2, and GIRK2 (Kir3.2) (Hansen et al., 2011; Whorton and MacKinnon, 2011). Based on these structures, PIP_2_ promotes an active conformation of the channels by inducing a translation of the cytoplasmic domain, both cytoplasmic and transmembrane domains being pulled together by the lipids. In TREK1 and TRAAK channels, the role of this lipid would be to anchor TREK1 pCt to the membrane, exerting a force to maintain the SF/M4 module in the “down” conformation in basal conditions. How TRAAK would be in the “up” conformation in basal conditions, with no or a weaker interaction of pCt with the membrane is an open question.

TREK and TRAAK channels regulate the resting membrane potential in many excitable and non-excitable tissues. However, basal channel activity in physiological conditions is not well established. From the phenotyping of KO mice, the level of activity of TREK1 seems to be dependent on the area of expression in the central nervous system. TREK1 KO mice are resistant to the TREK1 opener isoflurane and to the general anesthesia induced by this compound (Patel et al., 1998), suggesting that in wild-type mice TREK1 is not active in basal conditions and can be activated by isoflurane to produce anesthesia. On the other hand, the TREK1 KO mice are resistant to depression and behave like wild-type animals that have received the antidepressant fluoxetine (Patel et al., 1998) suggesting that in the wild-type mice TREK1 is open and can be blocked by fluoxetine. Based on these observations, the activity level of TREK1 is clearly not the same in neurons involved in anesthesia and in depression. Interaction with its partner proteins AKAP150 (Patel et al., 1998) and PLD2 stimulates their activity, and likely plays a role in controlling basal channel activity, but the interaction of pCt with PIP_2_, and potentially with other negatively charged phospholipids, may also be critical. Performing RNA sequencing of half a million of cells, a recent study has revealed the molecular architecture of the mouse nervous system (Zeisel et al., 2018). Neurons were mapped spatially and a hierarchical, data-driven taxonomy was derived. Mining of these data show that TREK1 and TRAAK have unique expression patterns in central and peripheral nervous systems, with neurons expressing only TRAAK, only TREK1 or various relative amounts of TREK1 and TRAAK. Given that TREK1pCt is dominant in the TREK1/TRAAK heterodimers, modulation of the respective expression levels of TREK1 and TRAAK will affect basal leak activity of these channels and input resistance of neurons, without changing the inhibitory current that can be generated by stimuli such as temperature, stretch or polyunsaturated fatty acids. Beside integration of stimuli, these polymodal channels would also regulate excitability through the dynamic regulation of their activity at rest.

## Materials and Methods

### Molecular Biology

Human TREK1 (KCNK2, genbank accession number AAH69462.1) and TRAAK (KCNK4, NCBI Reference Sequence: NP_201567.1) were cloned into pIRES2-eGFP vector (Clontech). All the chimeras and tandems were obtained by overlapping PCR and inserted into the same vector. Substitution of I292G293D294 in TREK1 and I253G254N255 in TRAAK by a repeat of glycines was performed by PCR using Pfu Turbo DNA polymerase (Agilent). All the constructs were verified by DNA sequencing.

### Cell Culture and Transfection

HEK cells were grown in 75-mm tissue-culture dishes (Falcon, Franklin Lakes, NJ) in Dulbecco’s modified Eagle’s medium (Gibco, Life Technologies, Saint Aubin, France) supplemented with 10% fetal calf serum (Hyclone, Thermo Fisher Scientific GMBH, Ulm, Germany) and 1% penicillin-streptomycin (Gibco, Life Technologies, Saint Aubin, France) in a humidified incubator at 37°C (5% CO2). For expression and electrophysiology of WT and mutant channels, 0,8 μg of plasmid was transfected using Lipofectamine (Life technologies, Grand Island, NY, United States) according to the manufacturer’s instructions. Cells were plated onto 35-mm dishes 24 h before transfection, and experiments were performed over the following 1–2 days.

### Electrophysiology

Pipettes were pulled from haematocrit-capillaries (Hirschmann Laborgeraete, Germany) using a vertical puller (PC-10, Narishige International, London, United Kingdom), and had resistances of 2–4 MΩ when filled with internal solution and measured in standard bath solution. Whole cell membrane currents were measured and filtered at 3 kHz by a RK 400 patch clamp amplifier (Bio-Logic Science Instruments), and digitized at 10 kHz using a 12-bit analog-to-digital converter Digidata-1322 (Axon Instrument, Sunnyvale, CA, United States). Recordings were done using Clampex 8.2 software (Axon Instrument). The external solution used to record TREK1 and TRAAK currents in HEK cells contained (in mM): 140 NaCl, 10 TEA-Cl, 5 KCl, 3 MgCl2, 1 CaCl2, 10 HEPES, pH 7.4, with NaOH. The pipette solution contained (in mM): 155 KCl, 3 MgCl2, 5 EGTA, 10 HEPES, pH 7.2, with KOH. In all experiments using VSP, the perforated-patch method of recording was used with Nystatin (600 ng/ml) in the pipette. Nystatin (Sigma-aldrich) was prepared as a stock solution as 60 mg/ml in DMSO. In these experiments, the access resistance was typically 7–10 MΩ 5–10 min after seal formation. Cells were placed in 35-mm dish through which solution flowed at 1–2 ml/min. Inflow to the dish was by gravity from several reservoirs, selectable by activation of solenoid valves (Warner Scientific). Bath solution exchange was essentially complete by <30 s. Experiments were performed at room temperature. Current decays were measured at +120 mV and then plotted as a function of time. Finally, the inhibition of the current by the VSP was measured by comparing current at 0 mV before and after activation of the VSP. Cell populations were compared using unpaired *t-*test. The data are given as the mean ± SE.

Single-channel recording was performed at cell-attached mode using a patch clamp amplifier (Axopatch 200; Axon Instruments). Single-channel currents were filtered at 2 kHz using an eight-pole Bessel filter (−3 dB; Frequency Devices) and digitalized with a Digidata 1320 interface (Axon Instruments) at a sampling rate of 20 kHz. Threshold detection of channel openings was set at 50%. Single-channel currents were analyzed with the pCLAMP 10 software. The filter dead time was 100 μs (0.3/cutoff frequency) for single-channel analysis. Pipette and bath solutions contained (in mM) 150 KCl, 1 MgCl2, 5 EGTA, and 10 HEPES (pH 7.3). The conductance and mean open time were obtained from amplitude and duration histograms, which were measured from channel that opens to only one conductance level at +80 and −80 mV.

### Live Cell Microscopy

The open reading frames of WT and mutant channels were fused in frame with the sequence of ecliptic pHluorin (pH) preceded by the sequence of the transmembrane domain of human transferrin receptor by overlapping PCR. The amplification products were inserted in pcDNA3 and verified by DNA sequencing. The corresponding chimeras were expressed in HEK cells grown on FluoroDishes by transient transfection using Lipofectamine 2000 following the manufacturer’s instructions. 24 h after expression, cell imaging was performed under an Ultraview Vox spinning disk confocal microscope (Perkin Elmer). Acquisition was performed with a 60×/1.40 objective using a 488 nm laser and a 527/55 dichroïc filter. Cells were imaged at 30 frames/min under perfusion of an extracellular medium at pH 7.4 (140 NaCl, 5 KCl, 3 MgCl2, 1 CaCl2, 10 HEPES, pH 7.4, with NaOH) or 6 (140 NaCl, 5 KCl, 3 MgCl2, 1 CaCl2, 10 MES, pH 6, with NaOH). Signal intensity was measured right before buffer changes under ImageJ using a home-made macro, and the results are expressed as the percentage of pH-sensitive fluorescence at pH 6 over the fluorescence at pH 7.4, after subtraction of the background. At the end of the experiments, cells were treated with ionophores (nigericin and monensin both at 10 μM) to equilibrate the pH of all intracellular compartments with the pH 7.4 medium.

## Author Contributions

FL conceived the experiments and wrote the manuscript. IBS, FSC, SB, E-JK, SF, FCC, DK, and DB contributed to the experimental work, conception of the experiments, and wrote the manuscript.

## Conflict of Interest Statement

The authors declare that the research was conducted in the absence of any commercial or financial relationships that could be construed as a potential conflict of interest.
