# Supplementary material for: Antagonistic Effect of a Cytoplasmic Domain on the Basal Activity of Polymodal Potassium Channels
Source: Front Mol Neurosci. 2018 Sep 4;11:301. doi: 10.3389/fnmol.2018.00301 (PMC6131555; doi:10.3389/fnmol.2018.00301)
Supplement: Supplementary file 1 [file Image_1.pdf]

## Supplementary Information

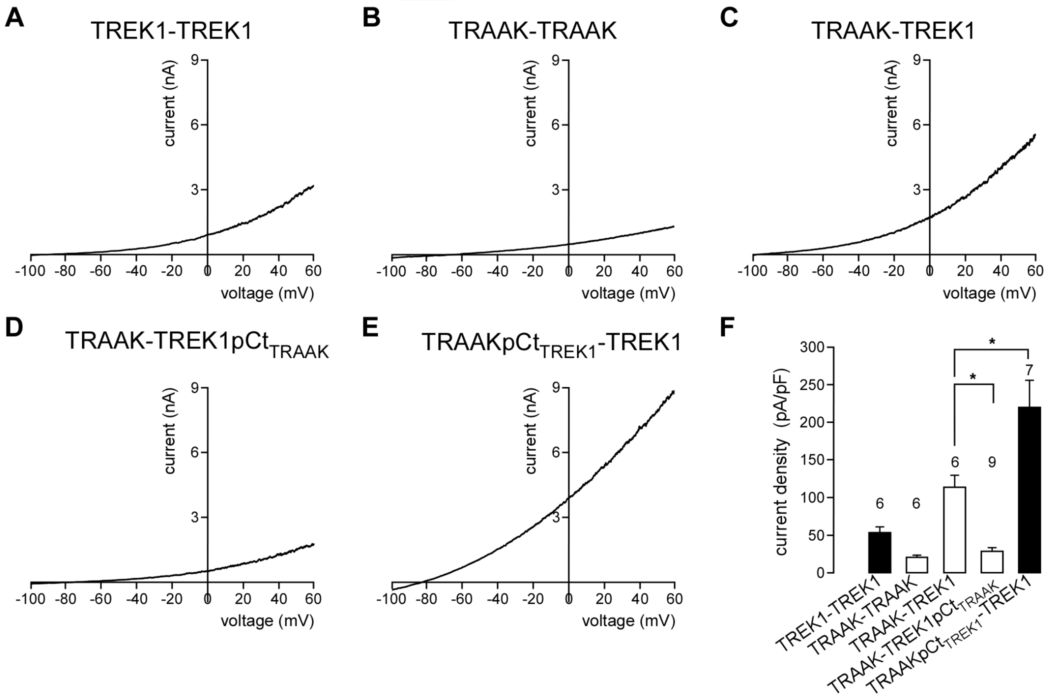


FIGURE S1. **pCt regulates current amplitude of the TRAAK-TREK1 heterodimer. A-E**, Representative whole-cell recordings from HEK cells expressing covalent tandems TREK1-TREK1 (**A**), TRAAK-TRAAK (**B**), TRAAK-TREK1 (**C**), TRAAK-TREK1pCt$_{TRAAK}$ (**D**) and TRAAKpCt$_{TREK1}$-TREK1 (**E**). **F**, Current densities at 0 mV for the indicated channels. Data are presented as mean ± SEM, *p<0.05, the number of cells is indicated (one-way Anova with Tukey's test).

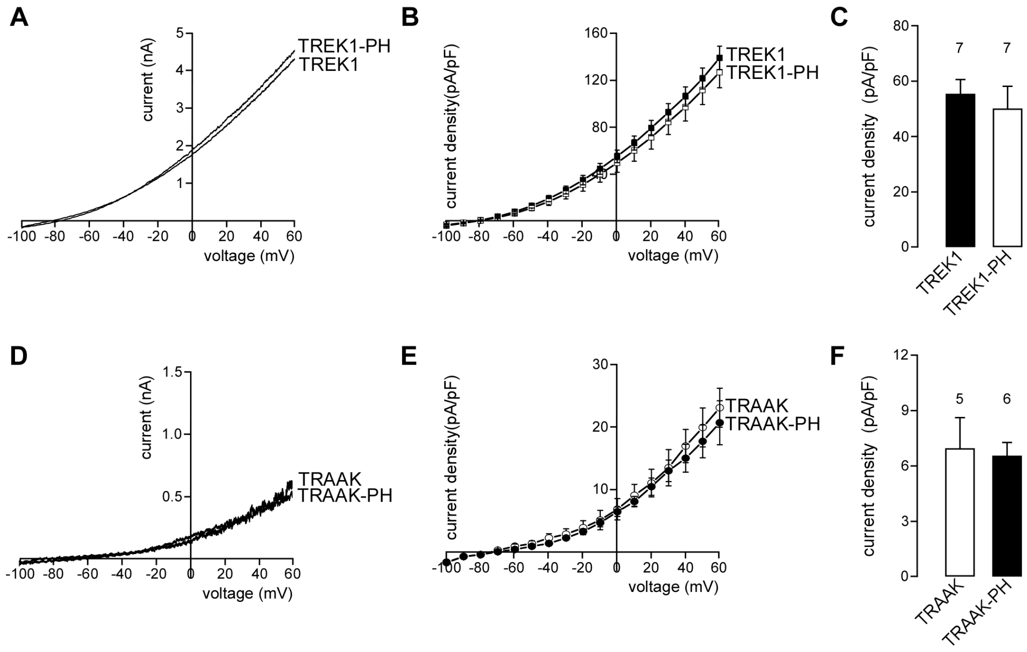


FIGURE S2. **Fusion of pHluorin to TREK1 and TRAAK does not affect channel activity. A, D**, Representative whole-cell recordings of HEK cells expressing TREK1 or TREK1-PH (**A**) and TRAAK or TRAAK-PH (**D**). **B, E,** Current density/voltage curves obtained from currents shown in A and D. **C, F**, Current densities at 0 mV, Data are presented as mean ± SEM. The number of cells is indicated (Student's t-test).

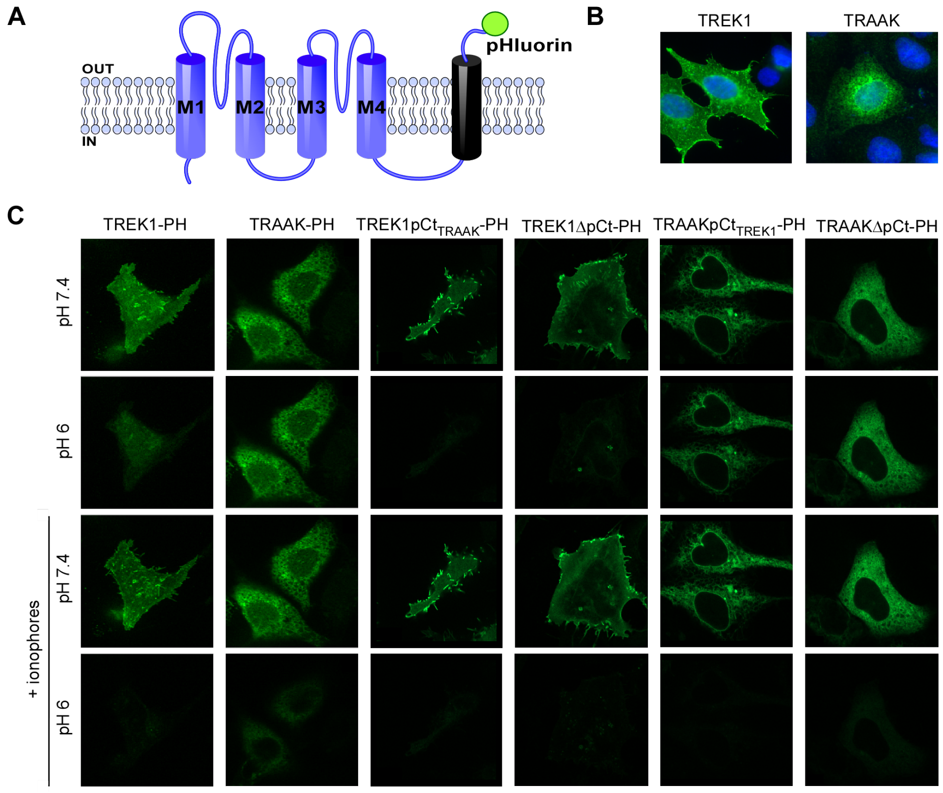


FIGURE S3. **Deletion or swapping of pCt does not affect channel cellular trafficking. A**, Schematic representation of pHluorin-tagged channels. An additional membrane-spanning segment was added between the channel and pHluorin, to expose pHluorin to the extracellular medium. **B**, Immunolabeling of wild-type channels expressed in HEK cells. Channels are in green. **C**, Representative images of pHluorin-tagged channels in live HEK cells. Acidification of the extracellular medium induces a quenching of pHluorin exposed at the plasma membrane without affecting fluorescence in internal cell compartments. Application of ionophores leads to the dissipation of the pH gradients, leading to a maximal signal at pH 7.4 and to a total fluorescence quenching at pH 6.

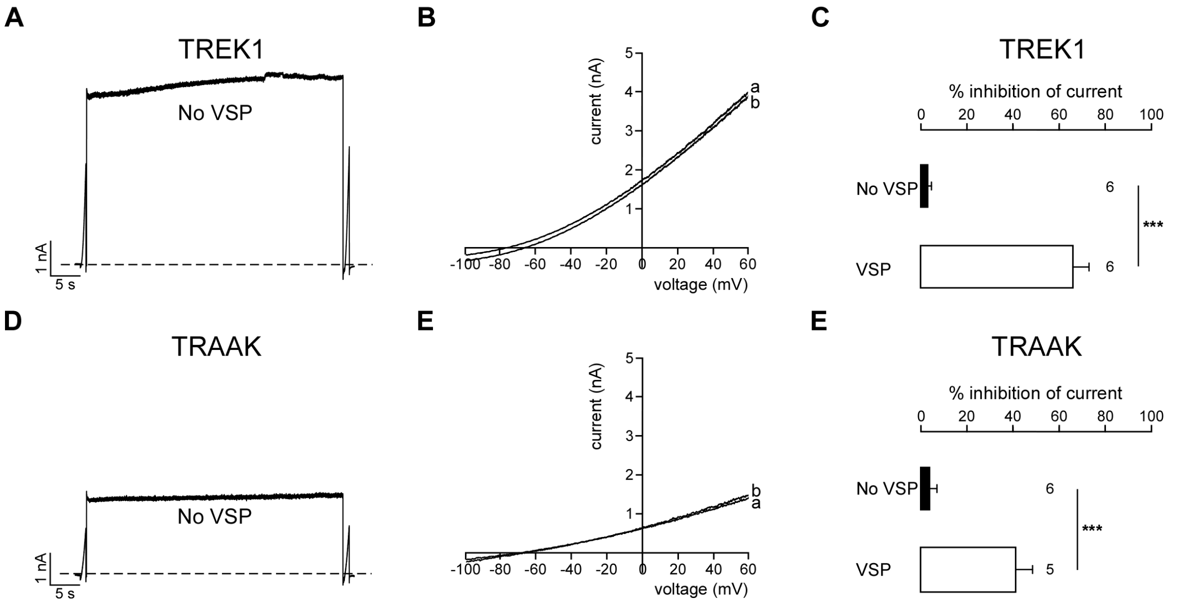


FIGURE S4. **TREK1 and TRAAK currents are not affected by strong depolarizations in the absence of VSP co-expression. A, D**, Representative perforated patch-clamp recordings from HEK cells expressing TREK1 (**A**) and TRAAK (**D**). Same protocol as in Fig. 6A and Fig. 7A**. B, E**, TREK1 (**B**) and TRAAK (**E**) currents before (a) and after (b) the +120 mV/40 s depolarizing pulse. **C**, **E**, Current inhibition (%) at 0 mV in cells expressing VSP *versus* cells not expressing VSP. Data are presented as mean ± SEM, ***p<0.001, the number of cells is indicated (Student's t-test).

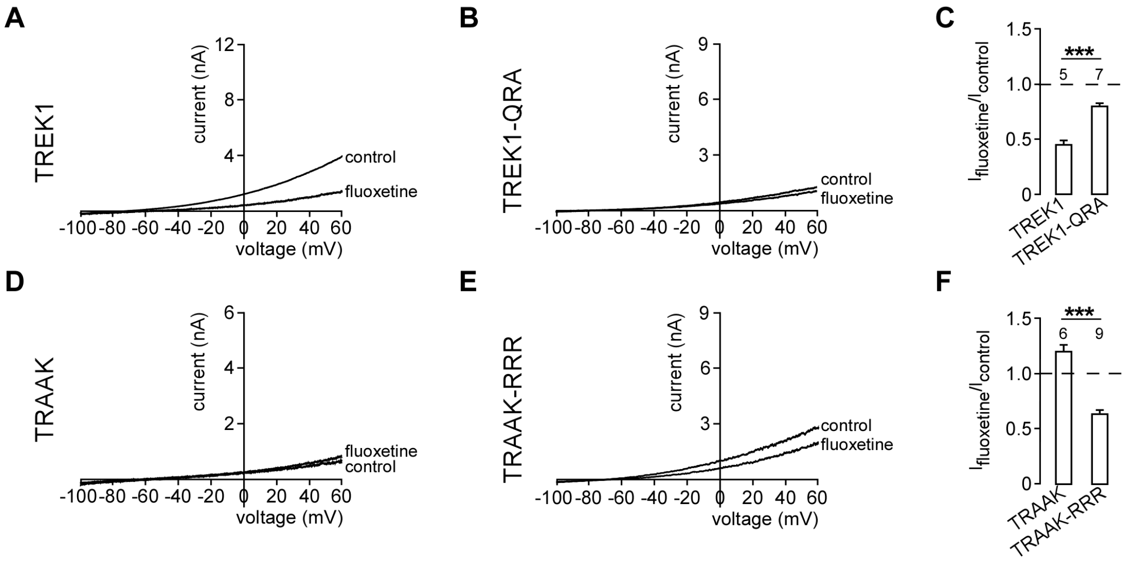


FIGURE S5. **A, B, D, E**, Representative patch-clamp recordings from HEK cells expressing wild-type (**A**, **D**) or mutant channels (**B**, **E**) in the presence of 10 µM fluoxetine on basal currents. **C, F**, Fraction of fluoxetine-sensitive current. Data are presented as mean ± SEM. ***p<0.001, the number of cells is indicated (Student's t-test).
